# Special Issue on “Disease and the Hippo Pathway”

**DOI:** 10.3390/cells8101179

**Published:** 2019-09-30

**Authors:** Carsten Gram Hansen

**Affiliations:** 1 University of Edinburgh Centre for Inflammation Research, Queen’s Medical Research Institute, Edinburgh bioQuarter, 47 Little France Crescent, Edinburgh EH16 4TJ, UK; Carsten.G.Hansen@ed.ac.uk; 2 Institute for Regeneration and Repair, University of Edinburgh, Edinburgh bioQuarter, 5 Little France Drive, Edinburgh EH16 4UU, UK

The Hippo pathway is a cellular signalling network, which plays major roles in organ homeostasis and development [1,2,3,4,5]. However, when this cellular signalling pathway is perturbed, diseases such as cancer, excessive fibrosis, metabolic disorders and impaired immune responses occur [1,2,3,4,5,6]. The current significant interest in the pathway continues to reveal new links between diseases and the Hippo pathway, as well as to provide new insights into what role the Hippo pathway plays in normal development, regenerative processes, organ size control [2,3,4,5], and cellular homeostasis, including fundamental processes such as cell size regulation [7,8]. This Special Issue provides an up-to-date overview of this exciting cellular signalling pathway.

The Hippo pathway is a serine/threonine kinase cascade that mediates the phosphorylation and, thereby, inactivation of the transcriptional co-regulators YAP and TAZ. A range of scaffolding proteins plays central roles in the dynamic regulation of this pathway. YAP/TAZ does not bind DNA directly and, therefore, utilizes transcription factors to mediate its transcriptional response. Phosphorylated YAP/TAZ is sequestered in the cytoplasm and subsequently does not bind to nuclear localised transcription factors [1].

In this special issue, Gundogdu and Hergovich authoritatively highlight the Mps one Binder (MOB) scaffolding proteins as central players. They further emphasize not only the disease relevance of the precise regulation of the MOBs and their direct involvement in the kinase regulation of the Hippo pathway, but also the MOBs’ important function in scaffolding other kinase complexes [9]. Next, Huh, Kim, Jeong, and Park analyse the regulation of the TEADs, the main transcription factors used by YAP/TAZ [10]. This is an exciting area of research that up until recently has been understudied. Targeting the transcription factors directly, instead of focusing on YAP/TAZ, could be a fertile avenue to pursue, especially considering the current challenges in targeting the Hippo pathway. Hillmer and Link then describe how YAP/TAZ modulates the chromatin through TEADs and a range of additional cofactors [11]. YAP/TAZ-mediated chromatin remodelling is a field that, due to recent technological advances, has revealed major mechanistic insights into how YAP/TAZ either activates or represses gene transcription. Hillmer and Link provide a concise overview of these recent developments and also summarise outstanding questions that need addressing in the years to come [11].

Luo and Yu then highlight the importance of G-protein-coupled receptors (GPCRs) as one of the main upstream regulators of the Hippo pathway [12]. Their discussion focuses on how mutations [13,14], as well as altered GPCR activity [13,15], might drive tumourigenesis in multiple tissues via elevated YAP/TAZ activity. Luo and Yu examine how this GPCR–Hippo axis can be targeted in cancer [12].

Rognoni and Walko (featured on the front page) discuss the importance of YAP/TAZ in skin physiology, including in wound healing processes [16]. The mammalian skin is a well-structured organ with distinct cell layers. The skin is therefore an intriguing model organ to study in the context of the Hippo pathway, as it highlights the importance of the mesoscale organisation and the context-specific temporal and spatial regulation of YAP/TAZ [16]. Wound healing that is not resolved causes excessive fibrotic scarring. Kim, Choi, and Mo follow on and continue the discussion on how dysregulated YAP/TAZ drives fibrosis in multiple organs and also elaborate on its consequences for cancer development and its therapeutic implications [17]. Most types of solid tumours have high YAP/TAZ activity, and the majority of these cancers appear addicted to YAP/TAZ hyperactivity [18,19]. Advanced prostate cancer is one of the leading cancers killing men worldwide. This prevalent cancer therefore urgently needs improved therapeutics [20]. Omar Salem and I detail the role of the Hippo pathway in prostate cancer. We interrogate how impaired Hippo pathway activity contributes to this deadly disease [21].

Tumour growth needs additional blood supply, as cancer cells require both nutrients and oxygen. Malignant progression is consequently often paralleled by an angiogenic switch, where the vasculature transitions from a quiescent to a proliferative state [22]. In addition, angiogenesis also facilitates metastasis. The role and importance of angiogenesis in cancer development and growth is therefore well established [22]. Angiogenesis is also important in a range of healthy processes, such as embryonic development and wound healing. Azad, Ghahremani, and Yang highlight YAP/TAZ’s critical roles in endothelial cells during angiogenesis not only in healthy but also in pathological processes, such as tumour vascular mimicry [23]. Brandt, North, and Link take advantage of the recent technical developments using CrispR to generate *lats2* knockout zebrafish. Interestingly, fish with somatic loss of function mutations of *lats2*, but not *lats1*, develop peripheral nerve sheath tumours [24]. The comparative low cost of maintaining zebrafish, the relative ease of genetic manipulations, the fast embryonic development, and the ability to carry out robust drug screens [25] will likely continue to make this a powerful model organism for research aiming at obtaining further fundamental insights into hyperactive-YAP/TAZ-driven human disease. Finally, Yamauchi and Moroishi [26] give an up-to-date and commanding overview of the Hippo pathway’s role in the adaptive immune system [27]. Upstream core kinase components play pivotal roles in adaptive immunity and in both pro- and anticancer immune responses. Interestingly, these processes occur both independently of as well as through YAP/TAZ regulation [27,28,29,30].

The pioneering discovery of the Hippo pathway in *Drosophila melanogaster* [31] and the subsequent recognition that the major pathway components are greatly conserved in mammals have firmly established the need for both powerful model organisms and in vitro cellular model systems as means to obtain detailed insights into fundamental biological processes driven by the Hippo pathway [1,2,3,4,5,6]. Recent discoveries reveal that a wide range of diseases are driven by dysfunctional Hippo pathway activity and drive the continued interest in the pathway [1,2,3,4,5,6]. The articles in this Special Issue written by leading experts cover a wide range of diseases that are driven by perturbed Hippo pathway activity. Research into the Hippo pathway is a fascinating field that continues to hold great promise, and which will undoubtedly produce further major discoveries in years to come.
